# A Cardiac Rehabilitation Program for Breast Cancer Survivors: A Feasibility Study

**DOI:** 10.1155/2021/9965583

**Published:** 2021-05-27

**Authors:** Filadelfiya Zvinovski, Julie A. Stephens, Bhuvaneswari Ramaswamy, Raquel E. Reinbolt, Anne M. Noonan, Jeffrey Bryan VanDeusen, Robert Wesolowski, Daniel G. Stover, Nicole Olivia Williams, Sagar D. Sardesai, Laxmi Mehta, Randi Foraker, Martha Gulati, Maryam Lustberg, Allison M. Quick

**Affiliations:** ^1^The Ohio State University Medical Center, Division of Medical Oncology Columbus, Columbus, OH, USA; ^2^The Ohio State University Center for Biostatistics, Columbus, OH, USA; ^3^The Ohio State University Medical Center, Division of Cardiology Columbus, Columbus, OH, USA; ^4^Washington University in Saint Louis, Center for Population Health Informatics, St. Louis, MO, USA; ^5^University of Arizona College of Medicine—Phoenix, Division of Cardiology, Phoenix, AZ, USA; ^6^The Ohio State University Medical Center, Department of Radiation Oncology Columbus, Columbus, OH, USA

## Abstract

**Purpose:**

The purpose of this study was to determine the feasibility and preliminary efficacy of a cardiac rehabilitation (CR) intervention in the breast cancer population.

**Methods:**

This single-arm feasibility study evaluated a 14-week CR intervention program in breast cancer survivors. Feasibility was defined as completion of at least 30/36 sessions of the program without serious adverse events (SAE) in 80% of patients. Secondary endpoints included the change in VO2 max, cardiovascular disease (CVD) risk factors, Duke Activity Secondary Index (DASI), Brief Fatigue Inventory (BFI), and QLQ-C30. All outcomes were reported as mean change and compared using paired *t*-tests.

**Results:**

A total of 25 patients were enrolled in the study. 18 patients of the 25 enrolled (72%) completed the 14 weeks program without SAE. The overall adherence to the study protocol was 60%. Of the 18 participants who did not withdraw from the program, 15 (83%) adhered to the study protocol and completed 30 or more sessions. There was a nonsignificant improvement in VO2 max (mean Δ0.5, *p*=0.6). The scores for DASI, BFI, and QLQ-C30 improved from baseline to posttreatment.

**Conclusion:**

A CR intervention in breast cancer survivors had high adherence in those who were able to complete the 14-week program. The program significantly improved patient reported physical activity, fatigue, and quality of life (QoL), without significant improvement in CVD risk factors. Implications for cancer patients are that early implementation of a CR program should be considered by practitioners as it improves QoL and exercise tolerance in breast cancer survivors.

## 1. Introduction

Cardiovascular disease (CVD) is the leading cause of death among women in the general population and among breast cancer survivors specifically [1, 2]. Increased utilization of mammographic screening and adjuvant therapy has improved the long-term survival of women with breast cancer. There are now more than 2.5 million female breast cancer survivors in the United States [1]. As more women survive longer, their risk of death from other causes has increased such that the majority of breast cancer survivors ultimately die of CVD rather than from cancer [3, 4]. Important risk factors such as physical inactivity, advanced age, obesity, and smoking are common to the etiology of both CVD and breast cancer [1–3]. A recent evaluation of National Health and Nutrition Examination Survey data highlighted the possible role of shared risk factors in the development of cancer, reporting that over 90% of cancer survivors have multiple CVD risk factors [4]. Achieving favorable changes in risk factors common to both CVD and cancer are associated with improved CVD and cancer survival, as well as lower cancer recurrence [5, 6]. Better strategies for managing and preventing CVD are needed for this population.

Evidence suggests that exercise also decreases long-term side effects from cancer treatments among breast cancer survivors and may provide additional physiological and psychological benefits [7, 8]. A program of regular exercise may also reduce levels of CVD risk factors and the resulting risk of future cardiovascular events [9, 10]. Existing studies only touched the surface of the feasibility of outpatient cardiac rehabilitation (CR) programs for the reduction of CVD risk among breast cancer survivors, especially those susceptible to the late effects of chest radiation and cardiotoxic cancer treatments such as trastuzumab, doxorubicin, and aromatase inhibitor therapy [3, 11].

A CR program is a unique model for providing an exercise intervention to breast cancer survivors. As per the statement from the American Heart Association for the need for effective and viable strategies to mitigate cardiovascular disease risk in cancer population, cardiac rehabilitation can play a role for potential solution. AHA statement provides an overview of the existing knowledge and rationale for the use of cardiac rehabilitation to provide structured exercise and ancillary services to cancer patients and survivors [12].

The CR program is defined as “the provision of comprehensive long-term services involving medical evaluation, prescriptive exercise, cardiac risk factor modification, and education, counseling, and behavioral interventions” [13]. CR is characterized by its evidence-based protocol and attention to exercise principles, including frequency, intensity, and duration [14], which is lacking in existing exercise studies [7, 15, 16]. CR programs are tailored exercise programs, which are modifiable according to the baseline cardiorespiratory fitness level and comorbid disease status of the participants and are designed to induce changes in the CVD risk profile of participants [17, 18].

Although CVD and breast cancer share many important risk factors, this current study is the first to study the implementation and feasibility of CR among breast cancer survivors posttreatment to improve cardiorespiratory fitness, reduce CVD risk, and improve quality of life (QoL). Earlier existing studies report increased CVD risk among breast cancer survivors [4] but have not yet assessed the feasibility of outpatient CR programs for the reduction of CVD risk among breast cancer survivors, especially those susceptible to the late effects of chest radiation and medical treatments. Treatments including drugs such as trastuzumab, doxorubicin, and most recently, aromatase inhibitor therapy are associated with this increased risk of cardiotoxicity [1, 11].

The purpose of this study was to assess the feasibility of a CR program in breast cancer survivors and to evaluate cardiorespiratory fitness, CVD risk factors, and patient reported outcome measures before and after the intervention.

## 2. Methods

This was a single-arm prospective feasibility study of a 36-session (14-week) CR intervention in female breast cancer survivors. A sample size of 20 participants was initially planned to evaluate the feasibility of the CR program. An additional five participants were accrued to account for attrition.

Participants' recruitment, eligibility screening, and consent occurred at the time of their appointment of their final breast cancer treatment and/or subsequent follow-up appointments. Women between the ages of 30 and 75 with stages 0–III breast cancer who were within 18 months of treatment including surgery, radiation, and/or chemotherapy, regardless of type and duration were eligible for the study. Patients could be receiving ongoing endocrine therapy or trastuzumab. Individuals with existing CVD, contraindications to exercise, or cardiac stress testing, metastatic breast cancer, other concurrent malignancies except skin cancer, active infection, psychiatric illness/social situation that would limit compliance with study requirements, or who were pregnant or breastfeeding were excluded from the study. An exercise prescription, approved per protocol by the CR program director and cardiac rehabilitation staff, was developed for each participant in the intervention group to guide the CR sessions.

This study was approved by the Ohio State University Medical Center Cancer Institutional Review Board and followed IRB guidelines.

### 2.1. Procedures

Demographics, clinical data, and inclusion/exclusion criteria were obtained and recorded for all patients. Variables of interest were participants' age, self-reported race/ethnicity, cancer treatment details, and medical history. Breast cancer patients typically progress through treatment in the following order: surgery, chemotherapy, and radiation therapy, though not all patients progress through each step. We recruited patients from their treatment close-out appointment to participate in the study, regardless of type or duration of therapy received. The rationale for the proposed research is that cardiac rehabilitation programs have substantial extant infrastructure and may improve morbidity and mortality from both CVD and cancer among breast cancer patients posttreatment.

The CR program consisted of one-hour sessions, three times per week for a maximum of 14 weeks and for a minimum total of 36 sessions. The program took place at an outpatient CR center at the Ohio State University Medical Center that was designated for cardiac patients. An exercise prescription, approved per protocol by the CR program director and cardiac rehabilitation staff, was developed for each participant to guide the CR sessions according to the cardiorespiratory fitness level of the participant. During each session, participants started the intervention at a workload of 60–85% of their VO2 max as determined in advance by a graded exercise stress test using the Bruce protocol [19]. The goal was to increase the duration of this workload to 45 minutes throughout the intervention period. Participants were encouraged to supplement their exercise program at home and to increase the frequency of exercise to five times per week.

A cardiac stress test was performed at baseline and week 14. The participants were instructed on the use of the Borg Perceived Exertion Scale for reporting their subjective level of exertion during the test [20]. The stress testing technician monitored the participants for symptoms such as chest pain or shortness of breath and ensured that the vital signs returned to normal following the test.

Patient questionnaires were administered at baseline, week 8, and week 14. Systolic and diastolic blood pressure (BP), a fasting lipid panel, fasting glucose, height and weight, and body mass index (BMI) of participants were checked at baseline and at the end of the 14-week intervention period.

During the 14-week intervention period, the CR staff contacted participants by phone to remind them of their upcoming CR appointments for the week. The participants received a $20 per week stipend for the 14-week intervention period to help offset travel costs to and from the CR facility. Upon completion of the 14-week assessment, all participants received an additional $50 stipend.

### 2.2. Measures

#### 2.2.1. Cardiorespiratory Fitness

Cardiorespiratory fitness was measured by the participants VO2 max in mL/kg/min at baseline and at the end of the 14-week intervention period. The VO2 max was directly obtained from all study participants using a face mask that measured the concentration of inhaled and exhaled gasses during a graded exercise stress test using the Bruce protocol [19]. During the test, the intensity of exercise was gradually increased. The participants' VO2 max was reached when the value of VO2 did not change with an associated increase in exercise intensity. If participant requested the graded exercise test to be stopped prior to reaching their VO2 max, their submaximal VO2 was recorded as their VO2 max. This test was performed on a treadmill. The graded exercise stress test protocol and related CR patient education classes including nutrition, weight training principles, medications, and heart disease followed the standard CR program. The participants' BP, heart rate, rate of perceived exertion (RPE) [20], and any symptoms including chest pain or shortness of breath were monitored before, during, and after the exercise.

#### 2.2.2. CVD Risk Factors

CVD risk factors including BP, heart rate (HR), BMI, fasting total low-density lipoprotein (LDL), total cholesterol, and fasting glucose were measured at baseline and at the end of the 14-week intervention period. The systolic and diastolic BP (mm/Hg) of each participant was assessed three times at one-minute intervals after the participant was seated with legs uncrossed for 5 minutes. The last two readings were averaged for the BP measurement. Height (cm) and weight (kg) were measured without shoes, and BMI (kg/m^2^) was calculated from these measurements.

### 2.3. Patient-Reported Outcomes

#### 2.3.1. Physical Activity

The Duke Activity Status Index (DASI) questionnaire was used to assess self-reported physical activity among all participants. The DASI is a 12-item questionnaire that assesses both activities of daily living and leisure-time physical activity. The summed score correlates with peak oxygen consumption [21]. Higher scores indicate higher functional capacity. The DASI has been shown to be valid, reliable, and independently prognostic of cardiac events in women [21].

#### 2.3.2. Quality of Life (QoL)

The European Organization for Research and Treatment of Cancer QoL core questionnaire (EORTC QLQ-C30) and its associated breast cancer module (EORTC QLQ-BR23) were used to measure QoL and were scored according to the EORTC scoring manual [22]. The EORTC questionnaires are validated tools designed to assess QoL among cancer patients across several dimensions, including global health status, social functioning, fatigue, nausea and vomiting, pain, dyspnea, insomnia, loss of appetite, constipation, diarrhea, and financial strain. The QLQ-BR23 expands the functional scales to include body image, sexual functioning, sexual enjoyment, breast symptoms, arm symptoms, and hair loss. The EORTC questionnaire was self-administered with paper and pencil. If the participants missed their 8-week visit or did not attend their 14-week visit, the EORTC questionnaire was administered by phone or mail.

#### 2.3.3. Fatigue

The Brief Fatigue Inventory (BFI) questionnaire was used to measure self-reported fatigue. BFI is an instrument for evaluation of fatigue and its impact on daily life in patients with cancer. The BFI is a short, validated measure of fatigue severity which complements findings from the EORTC questionnaires [23].

#### 2.3.4. Participant Satisfaction

Participants were asked to rate their degree of satisfaction with the exercise portion of the program, the education portion of the program, and the overall cardiac rehabilitation experience on a scale of 1–10, with 1 being extremely dissatisfied and 10 being extremely satisfied.

#### 2.3.5. Adverse Events

Adverse events were reported using the Common Terminology Criteria for Adverse Events v4.0 (CTCAE) of the National Cancer Institute. Grade 3, grade 4, and grade 5 toxicities were reported as adverse events.

### 2.4. Statistical Analysis

The primary endpoint of the study was to assess the feasibility of conducting a 14-week CR program in women with breast cancer after completion of therapy. Feasibility was defined as completion of at least 30 sessions of the program without serious adverse events (SAE) in 80% of patients and was reported as a percent with 95% confidence interval.

Secondary endpoints of the study included the change in cardiorespiratory fitness and DASI. Tertiary endpoints included CVD risk factors, QoL, and patient satisfaction at baseline and 14 weeks.

Summary statistics for patient demographics and disease characteristics were calculated. The proportion of participants who completed the CR program was calculated, along with the 95% confidence interval (CI). The change in each endpoint from baseline to 14 weeks was calculated and summarized by mean, standard deviation, and 95% CI. The difference in these outcomes from baseline to 14 weeks was compared using either a two-sample *t*-test or sign-rank test. All data analyses were performed using SAS 9.4 (SAS Institute Inc., Cary, NC).

## 3. Results

### 3.1. Feasibility

**Figure 1:**
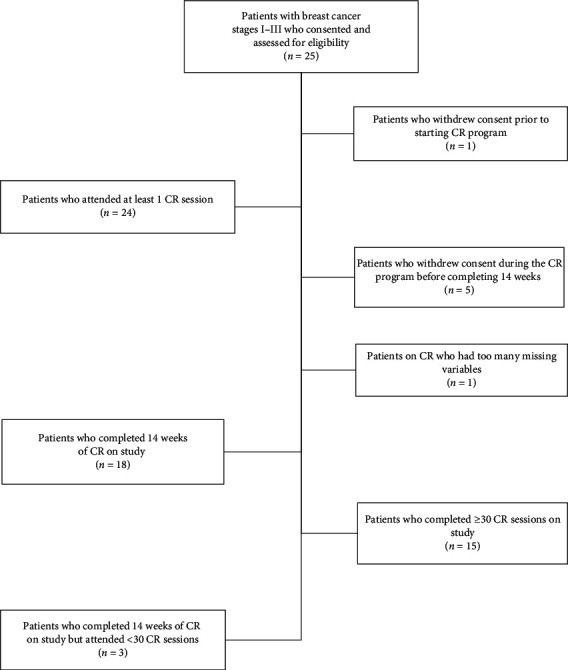
Consort diagram.

Twenty-five participants were consented and enrolled in the study (Figure 1). Participant characteristics are summarized in Table 1 and cancer characteristics are summarized in Table 2. The median age was 53 (range: 35–71), and the majority of women had ER/PR positive/Her2-neu negative (*n* = 13, 54%), stage I (*n* = 13, 54%) or II (*n* = 7, 30%) breast cancer.

Seventy-two percent (*n* = 18) of those participants who enrolled completed the CR program (95% CI: 50.6, 87.9). Seven participants withdrew from the study. One patient could not tolerate the schedule, one did not want to start the program, two developed an arrhythmia during the program and were unable to return, two withdrew consent, and one did not return for the program for undocumented reasons. 60% of those enrolled in the study (95% CI: 42.5, 82.0) completed 30 or more sessions and adhered to the study protocol. Of the 18 participants who did not withdraw from the CR program, 83% (*n* = 15) adhered to the study protocol and completed 30 or more sessions without SAEs.

### 3.2. Cardiorespiratory Fitness and CVD Risk Factors

**Figure 2:**
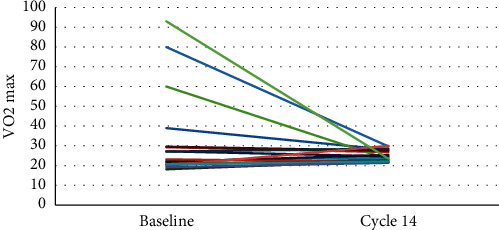
Change in VO2 max from baseline to cycle 14. Each individual color represents a different participant and their change in VO2 max from baseline to end of study at cycle 14.

Secondary outcomes included the change in cardiorespiratory fitness and CVD risk factors. There was a modest improvement in the VO2 max from the start of CR program to 14 weeks follow-up (mean Δ0.5, 95% CI: −20.0, 5.6, *p*=0.587) (Figure 2).

There was also some improvement in CVD risk factors including HR (Δ0.95, 95% CI: −2.8, 4.7, *p*=0.595), systolic BP (Δ1.76, 95% CI: −5.5, 9.0, *p*=0.612), and diastolic BP (Δ5.18, 95% CI: −0.5, 10.9, *p*=0.071) between baseline and 14-week follow-up. Improvements were also found in BMI (Δ−0.14, 95% CI: −0.9, 0.7, *p*=0.714), fasting blood glucose (Δ−0.53, 95% CI −5.4, 4.34, *p*=0.821), total cholesterol (Δ−4.71, 95% CI: −23.1, 13.7, *p*=0.589), and LDL cholesterol (Δ−4.93, 95% CI: −22.8, 12.9, *p*=0.561. Although these secondary and tertiary outcomes did show improvement, the study was not powered to detect statistical significance.

### 3.3. Patient-Reported Outcomes

**Figure 3:**
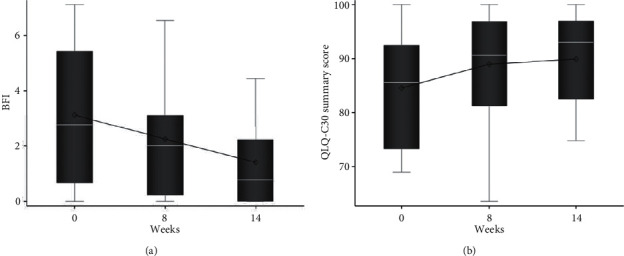
Box-plot showing the summary statistics for BFI and QLQ-C30 summary score by cycle. Mean (diamond), median (white line), and the 1^st^ and 3^rd^ quartiles (Q1: bottom and Q3: top of box) are displayed. The whiskers are the distance equal to 1.5 times the interquartile range (IQR) from Q1 and Q3. The diagram also shows outliers (circles) which are values that are above or below the whisker ends (Q1 − 1.5∗ IQR and Q3 + 1.5∗ IQR).

There was improvement in the patient reported physical activity according to the DASI (mean Δ13.2; *p* < 0.001), mean BFI score (mean Δ−1.7; 95% CI: −2.9, −0.5, *p*=0.007), and QLQ-C30 (Δ5.66; 95% CI: 1.68, 9.63, *p*=0.008) scores during the treatment period.  Figure 3 shows the changes in BFI and QLQ-C30 score at baseline, 8-week follow-up, and 14-week follow-up. Patients reported improvement in their fatigue and quality of life on the BFI (*p*=0.007) and QLQ-C30 (*p*=0.008) as early as at the 8-week study period. On the BR23 scales, participants reported improvement in body image, sexual functioning, breast symptoms, and systemic therapy side effects over the 14-week period.

### 3.4. Patient Satisfaction

Of those 18 participants who remained in the CR program until the end of the 14 weeks, 15 participants (83.3%) rated they were very to extremely satisfied, with the majority (*n* = 12, 67%) reporting extreme satisfaction, with the exercise portion of the program. Most of the participants (*n* = 11, 1%) were also very to extremely satisfied with the education component of the program, and 15 participants (83.3%) were very to extremely satisfied with the overall CR experience.

### 3.5. Adverse Events

Two patients withdrew from the study due to serious adverse events related to cardiac arrhythmias. One patient experienced ventricular tachycardia (grade 4) and required cardiac catheterization during the study period and could not continue with the study protocol past week 10. Another patient experienced supraventricular tachycardia (SVT), and the patient could not return to the study protocol following treatment for the SVT (grade 3). These events did not occur during the CR sessions, but are possibly related to the study intervention. Moreover, one patient experienced a headache during the study intervention (grade 1).

## 4. Discussion

The breast cancer survivors who successfully completed the 14-week CR intervention have shown significant improvement in patient reported physical activity, fatigue, and quality of life (QoL), but without significant improvement in CVD risk factors. Our long-term goal is to decrease the burden of CVD among breast cancer survivors. Moreover, with our central hypothesis, the cardiac rehabilitation intervention will improve cardiorespiratory fitness, CVD risk factors, and QoL among survivors. To mitigate CVD risk in cancer patients, there is a need of effective strategies, and the use of a cardiac rehabilitation (CR) programs has been significantly helpful in selected patients. The cardiooncology rehabilitation (CORE) model has been used to identify patients at high risk of CVD including cardiotoxicity due to cancer therapies [12]. Recent evidence indicated that a CR program is clinically beneficial and cost effective; also, the quality of delivery of a CR program is associated with the morbidity profile of patient population, and the role of exercise, physical activity, and nonpharmacological treatments are proven as a preventive measure for cardiovascular toxicity and modified cardiovascular risk in cancer survivors [24–28].

It is estimated that nearly 90% of women diagnosed with breast cancer will be alive in 5 years, which has important implications for CVD morbidity and mortality [29]. Unfortunately, the effects of breast cancer treatment are known to cause cardiotoxicity [30]. For example, anthracycline therapy, which is often a component of neoadjuvant and adjuvant therapy for high-risk breast cancer, can cause dose-dependent cardiotoxicity that leads to left ventricular dysfunction, congestive heart failure [31]. Trastuzumab can increase the risk of heart failure and left ventricular ejection fraction decline [32]. Radiation therapy to the breast or chest wall has been associated with dose-dependent cardiotoxicity which leads to radiation-induced fibrosis of the myocardium and microvascular damage to the coronary vasculature that accelerates atherosclerosis and development of coronary artery disease [33]. As immunotherapy becomes part of the standard of care for breast cancer treatment, the cardiotoxic effects of monoclonal antibody-based or targeted kinase therapies may contribute to the problem [34]. The shared risk factors combined with cardiotoxicity from breast cancer treatments increase the susceptibility of developing CVD, known as the “multiple-hit” hypothesis [35].

A recent report indicates that older female breast cancer survivors were more likely to die of CVD than breast cancer. Moreover, those patients that had comorbid conditions, such as CVD, had similar or worse survival compared to survivors with a higher stage of breast cancer without these comorbid conditions [36]. Early recognition of patients who are at risk of cardiac toxicity from their treatment and other risk factors for cardiac disease is essential in improving outcomes for breast cancer survivors [37].

The primary objective of this study was to assess the feasibility of a CR program in the breast cancer population and is one of the first studies to prospectively assess the feasibility of a CR regimen in this population. Although only 60% of those enrolled on the study adhered to the protocol, 83% of those who completed the CR program were able to adhere to the program and complete 30 or more sessions. This rate is similar to an average completion rate of 60% for the general CR population [38]. The findings are also consistent with completion rates in other studies of cardiac rehabilitation in cancer survivors and are much higher than that reported in a prospective study of breast cancer survivors using a community CR program who had an adherence rate of 30% [39]. Consistent with a previous retrospective study of CR in breast cancer patients that found an increase in VO2 max in participants [40], we found a nonsignificant increase in VO2 max in participants from baseline to the end of the program. As Table 3 demonstrates, other non-CR based exercise interventions have also demonstrated a benefit of exercise on VO2 max for women with breast cancer by attenuating the effects of chemotherapy during breast cancer treatment and by allowing recovery of cardiopulmonary function after the completion of treatment for breast cancer [46]. The study also found that participant' self-reported physical activity increased based on the DASI during the program, which is consistent with other studies that have shown an increase in cardiopulmonary fitness and overall physical function after an exercise program based on the DASI [49].

There have been conflicting results on changes in cardiac markers from a CR program. One study did not demonstrate an improvement in heart rate and blood pressure from baseline to postintervention [45], while another study showed normalization of BP and lowering of HR with an exercise program [48, 50]. A previous study observed a significant improvement in circulating biomarkers such as insulin levels after exercise training in obese breast cancer survivors [43]. The current study is the first study to look at changes in fasting glucose, cholesterol, and BMI during an exercise program in breast cancer patients. There was a decrease in cholesterol and glucose after completion of the CR program, but the change was not significant. However, since the study was a feasibility study, it was not powered to detect a difference between the values for these endpoints.

A CR platform is known to improve the quality of life for patients that complete the program, including improvement in fatigue [41, 47]. Several studies have also shown similar benefits in QoL in breast cancer patients undergoing exercise training [44, 51]. Our study found that there is a significant improvement in BFI and QLQ-C30 over the intervention period and at follow-up, supporting the previous evidence that fatigue and QoL improve after a CR program [41, 47].

### 4.1. Strengths and Limitations

The strength of this study was that it used an existing outpatient CR program, including its convenience to hospital-based services and treatments, which have been shown to be efficient and cost effective from a health services perspective [42]. Most private insurance carriers and medicare reimburse for CR utilized for cardiac conditions but not for cancer survivors, given current lack of evidence. However, unlike many exercise programs, CR is characterized by its evidence-based protocol and attention to exercise principles. The AHA released a statement in 2019 emphasizing the importance of CR in the cancer survivor population [12]. Additional strengths of the study include the broad eligibility criteria, which is more representative of the general population of breast cancer survivors. Moreover, the use of objective measures such as VO2 max, vital signs, biometric studies (such as cholesterol and fasting glucose), and the use of validated questionnaires strengthened the study [21–23]. Furthermore, the study is one of a few prospective CR intervention studies for breast cancer survivors.

The limitations of the study include the follow-up at only 8 and 14 weeks from the last treatment. Further investigations with longer-term follow-up are needed to assess duration of the positive effects of the program and to further evaluate changes in cardiac risk factors. Another limitation of the study includes the overall general health of the participants and lack of significant comorbidities in the CR program. Unfortunately, many patients in the oncology population have other comorbidities that would modify their CR program prescription. Moreover, the satisfaction results with the CR intervention are limited due missing data from the people who did not complete the program. Additional important limitations of our study include the lack of a placebo comparison arm, lack of blinding, and no treatment randomization. Therefore, the next step is to complete a double-blind, randomized controlled trial with significantly larger study population, comparing VO2 max in patients undergoing CR program to current standard of care follow-up, which does not include an organized exercise program.

## 5. Conclusions

Taken together, our study suggests that CR program in breast cancer survivors is feasible but does not meet is predefined criterion of feasibility. Adherence to the CR program among breast cancer survivors was similar to the general population. While a CR intervention in breast cancer survivors showed a modest improvement in VO2 max and CVD risk factors, it did demonstrate an improvement in patient reported outcomes of increased physical activity, fatigue, and QOL, in a significantly limited period of time suggesting that a larger randomized clinical trial would enroll much higher patient number that should be undertaken.

## Figures and Tables

**Table 1:** Summary of demographic and screening variables.

Variable	Level	Total (*n* = 24)
Age		
	Median (IQR) (min, max)	53 (46, 60) (35, 71)
Race		
	Asian	1 (4%)
	Black or African American	2 (8%)
	White	21 (88%)
Ethnicity		
	Non-Hispanic	24 (100%)

**Table 2:** Summary of screening tumor characteristic variables.

Variable	Level	Total (*n* = 24)
Breast cancer staging (overall)		
	IA	12 (50%)
	IB	1 (4%)
	IIA	4 (17%)
	IIB	3 (13%)
	IIIA	2 (8%)
	IIIB	2 (8%)
# of nodes		
	0	18 (75%)
	1	2 (12%)
	2	2 (8%)
	6	1 (4%)
	8	1 (4%)
History of prior cardiac events, previous diagnosis of hypertension, hypercholesterolemia, or diabetes?		
	Missing	1 (4%)
	No	22 (92%)
	Yes	1 (4%)
Estrogen receptor		
	Negative	4 (17%)
	Positive	20 (83%)
Progesterone receptor		
	Negative	6 (25%)
	Positive	18 (75%)
Her2-neu (IHC)		
	0	9 (38%)
	1	8 (33%)
	2	2 (8%)
	3	5 (21%)
Her2-neu (FISH)		
	Negative	17 (71%)
	Positive	7 (29%)
ER/PR/Her2-neu		
	Missing	1 (4%)
	ER+/PR+/HER2+	4 (17%)
	ER+/PR+/HER2−	13 (54%)
	ER+/PR−/HER2+	1 (4%)
	ER+/PR−/HER2−	1 (4%)
	ER−/PR−/HER2+	2 (8%)
	ER−/PR−/HER2−	2 (8%)
Grade		
	1	3 (12%)
	2	10 (42%)
	3	11 (50%)
Location of primary breast tumor		
	Bilateral	1 (4%)
	Left	12 (50%)
	Right	11 (46%)
Tumor size		
	Median (min, max)	1.5 (0.4, 3.8)

**Table 3:** Summary of current research on cardiac rehabilitation in breast cancer patients.

Author	Sample size (*n*)	Methods	Results
Battaglini et al. [41]	51 randomized controlled trials	Meta-analysis of studies including aerobic exercise, resistance programs, and combination of both.	Improvements in cardiorespiratory function, body composition, strength, and patient reported outcomes including fatigue, depression, and quality of life.
Bland et al. [42]	*n* = 68	Women with early stage breast cancer receiving chemotherapy participated in supervised aerobic and resistance exercise was prescribed three times per week during treatment, then one to two times per week for 20 additional weeks.	Higher baseline quality of life (QoL) predicted higher attendance during chemotherapy and higher QoL, measured at the end of treatment, and predicted higher attendance posttreatment.
De Jesus et al. [39]	*n* = 24	Feasibility study looking at a 16 week CR prescription program for breast cancer patients who rated their fatigue >4/10 after completion of adjuvant chemotherapy.	Adherence rate to exercise program was 30.3% to the cardiac rehab program. Improvements were seen in fatigue. No significant changes in body composition, aerobic exercise capacity, and activity patterns.
Dieli-Conwright et al. [43]	*n* = 100	Randomized controlled trial with 16-week combined aerobic and resistance exercise training in ethnically diverse sedentary, overweight and obese survivors of breast cancer	Sarcopenic obesity, circulating biomarkers (insulin, leptin, and adiponectin) significantly improved postintervention at 3-month follow-up
Dieli-Conwright et al. [44]	*n* = 200	Breast cancer survivors with sedentary lifestyles who are obese or overweight participated in a 16-week aerobic and resistance exercise training.	At postintervention, the exercise group was superior to usual care for quality of life, fatigue, depression, estimated VO2 max , muscular strength, osteocalcin, and bone specific alkaline phosphatase.
Dolan et al. [45]	*n* = 152	Retrospectively analyzed eligible charts of patients that participated in weekly supervised personalized aerobic and resistance exercise session for 22 group sessions plus 12 group educational sessions	Cardiorespiratory fitness (VO2 peak) improved by 14% with significant improvements in quality of life and depression scores.
Howden et al. [46]	*n* = 28	Patients with early stage breast cancer undergoing anthracycline therapy chose exercise training or usual care. The exercise training group completed 2 × 60 minute supervised exercise sessions per week.	Exercise training attenuated the VO2 decrease during chemotherapy. Functional disability can be prevented with exercise training.
Hsieh et al. [40]	*n* = 96	Patients in individually supervised oncology rehabilitation setting based on CR. 2-3 times/wk both aerobic and resistance and stretching.	↑ VO2 max and time on treadmill for all groups.
Juvet et al. [47]	*n* = 3418 25 randomized controlled trials	Systemic review of trials with physical exercise intervention versus a control group.	An increase in physical functioning and a decrease in fatigue were observed after a physical exercise intervention.
Kirkham et al. [48]	*n* = 73	Patients received adjuvant chemotherapy participated to varying degrees in supervised aerobic and resistance exercise during chemotherapy +/- radiation and for 20 weeks.	Chemotherapy resulted in increased HR_rest_ and tachycardia, decreased blood pressure. Anthracyclines, trastuzumab, and left‐sided radiation were associated with HR_rest_ elevations and impairments of HR_recovery_, but exercise training at least twice a week mitigated these changes.
Knobf et al. [49]	*n* = 154	Randomized controlled trial. Compared 2-month aerobic-resistance fitness center intervention to home-based physical activity.	Fitness center intervention had significantly improved time on treadmill, improved heart rate recovery at 1 min, greater MET minutes/week, a trend for improved insulin resistance, and stable insulin levels compared to the home-based physical activity group.
Lee et al. [50]	*n* = 4980	Analysis and multivariable linear regressions were used to examine the association between resting heart rate and metabolic risk factors, including systolic blood pressure, diastolic blood pressure, glucose, triglyceride, total cholesterol, high-density lipid cholesterol, and low-density lipid cholesterol in breast cancer survivors.	Strong positive association of resting heart rate with fasting glucose, triglycerides, and diastolic blood pressure in breast cancer survivors
Mutrie et al. [51]	*n* = 203	Patients in a 12-week supervised group exercise program during treatment for early stage breast cancer, with six-month follow-up.	Functional and psychological benefit after 12 weeks and six months later. No improvement in general scale of QoL (FACT-G). Improvement in mood and cancer specific QoL scale (FACT-B).

## Data Availability

The patient collection data used to support the findings of this study are available from the corresponding author upon request.
